# Targeting nucleolin-driven cancer stemness suppresses high-risk neuroblastoma *in vivo*

**DOI:** 10.1016/j.gendis.2026.102278

**Published:** 2026-06-06

**Authors:** Dan Qi, Ronghu Ke, Euni Wu, Yizhong Pan, S. Stephen Yi, Jason H. Huang, Erxi Wu

**Affiliations:** a Department of Neurosurgery and Neuroscience Institute, Baylor Scott & White Health, Temple, TX 76508, USA; b Baylor Research Institute, Baylor Scott & White Health, Temple, TX 76508, USA; c Department of Neurosurgery, Baylor College of Medicine, Temple, TX 76508, USA; d Department of Electrical and Computer Engineering, Cockrell School of Engineering, The University of Texas at Austin, Austin, TX 78712, USA; e Naresh K. Vashisht College of Medicine and Irma Lerma Rangel College of Pharmacy, Texas A&M University, College Station, TX 77843, USA; f Cancer Research Center and Department of Internal Medicine, Dell Medical School, The University of Texas at Austin, Austin, TX 78712, USA

Neuroblastoma (NB) is the most common extracranial solid tumor of childhood and arises from neural crest-derived cells of the developing sympathoadrenal lineage, most frequently in the adrenal glands. Notably, neural crest-derived cells intrinsically possess stem-like properties, including multipotency and developmental plasticity, which are partially retained in NB. Clinically, NB spans a broad spectrum from spontaneously regressing tumors to high-risk NB, which comprises ∼50% of cases and accounts for most disease-related mortality. Despite aggressive multimodal therapy—including intensive chemotherapy, surgery, radiation, stem cell transplantation, and immunotherapy—event-free survival for high-risk NB remains ∼40%–50%, and relapse is typically fatal.^1^ Thus, identifying actionable determinants that drive relapse and therapeutic resistance remains a critical unmet need in NB.

A defining feature of NB, particularly high-risk NB, is its profound intratumoral heterogeneity and tumor cell-state plasticity, including adrenergic and mesenchymal programs that can interconvert under therapeutic pressure. Importantly, the mesenchymal state exhibits enhanced stem-like characteristics and is associated with therapy resistance, immune evasion, and relapse propensity, limiting the durability of targeted therapies.^2^ In parallel, converging evidence demonstrates that cancer stem cells (CSCs) characterized by markers such as CD34, c-Kit, and nestin, exhibit self-renewal capacity and tumor-initiating ability, and contribute to metastasis, treatment resistance, and disease recurrence in NB.^3^ These findings underscore CSCs as central barriers to durable responses to current therapies in NB. However, clinically relevant regulators that link CSC maintenance with aggressive disease behavior *in vivo* remain poorly defined.

Nucleolin (NCL), a multifunctional RNA/DNA-binding nuclear protein, is increasingly linked to cancer stemness and plasticity, but its functional and prognostic significance in NB remains insufficiently defined. Seminal work from our group established a direct mechanistic role for NCL in maintaining NB stemness, demonstrating that NCL regulates CD34^+^ CSCs by binding to their promoter regions and modulates sensitivity to anti-CSC agents such as salinomycin.^4^ Since this initial report, accumulating evidence across multiple cancer types has established NCL as a central regulator of CSC programs through its roles in transcriptional regulation, cellular plasticity, and stress–response pathways.^5^ Nevertheless, prior studies, including our own, have largely not addressed the prognostic value of NCL in aggressive and relapsed NB patients.

In prior work, we showed that elevated NCL was associated with worse overall and event-free survival in a 498-patient NB cohort, with validation in an independent 649-patient cohort.^4^ We also observed a positive correlation between NCL expression and advanced International Neuroblastoma Staging System (INSS), with significantly higher expression observed in stage 3 and 4 tumors compared with lower-stage disease in the 649-patient NB cohort in our previous report. In the present study, this association was further confirmed in the 498-patient cohort (Fig. S1A). Cox regression in this cohort further showed that NCL was more strongly associated with overall survival than MYCN, ALK, and TERT (Fig. S1B), which are established NB biomarkers present in subsets of patients.

We next extended our analysis of NCL in the TARGET cohort (*n* = 249), with a focus on biologically aggressive, high-risk, and relapsed disease subsets. High NCL expression predicted inferior survival in high-risk patients (*n* = 217; Fig. 1A), with a more pronounced effect in patients with progressive or relapsed disease (*n* = 67; Fig. 1B). Consistently, NCL expression was higher in high-risk patients in the 498-patient cohort (Fig. 1C) and in patients with disease progression or recurrence in an independent 88-patient cohort (Fig. 1D). Within the TARGET cohort, the hazard ratio for NCL expression was 1.8 (*p* < 0.001) among high-risk patients overall and rose to 3.1 (*p* = 0.0018) in patients who had progressed or relapsed and whose tumors were undifferentiated or poorly differentiated (Fig. 1E). Additionally, NCL expression was higher in MYCN-amplified tumors, whereas Cox regression analyses showed that NCL exhibited higher hazard ratios than ALK and TERT (Fig. S1C and S1D). High NCL expression was also observed in non-MYCN-amplified patients. Together, these data establish NCL as an independent prognostic marker that identifies a biologically aggressive, relapse-prone subset of NB, with effects observed across both MYCN-associated and MYCN-independent contexts.

**Figure 1:**
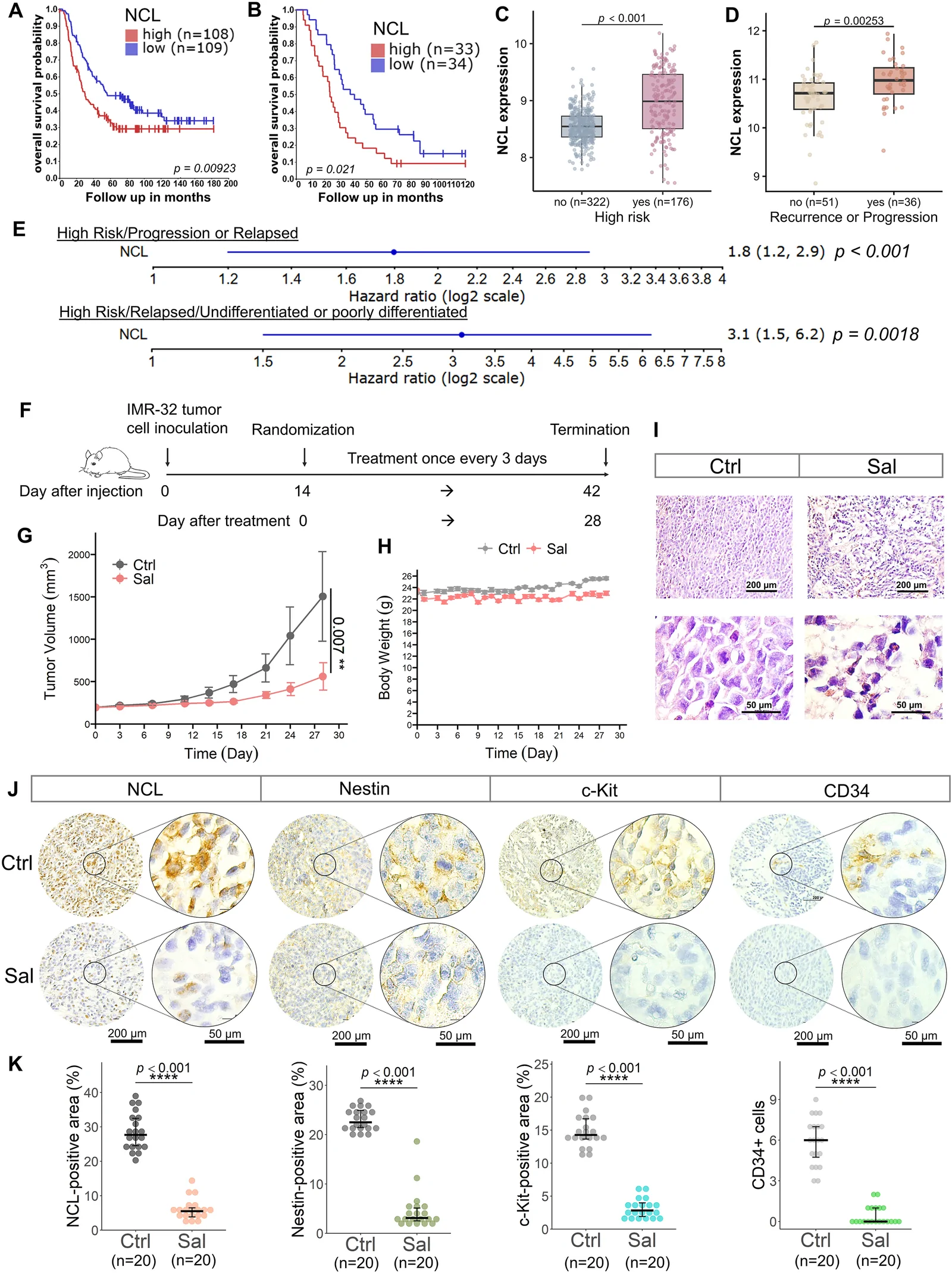
High NCL expression defines aggressive, relapse-prone NB and pharmacologic targeting of NCL suppresses tumor growth and CSC markers *in vivo*. **(A, B)** Kaplan–Meier analyses of overall survival in the TARGET NB cohort stratified by high versus low NCL expression (median cutoff). (A) Overall survival in a high-risk subset of the TARGET NB cohort (high NCL, *n* = 108; low NCL, *n* = 109). (B) Overall survival in progression and relapsed disease subsets of the TARGET NB cohort (high NCL, *n* = 33; low NCL, *n* = 34). Exact *p* values are reported. *p* values were calculated using the log-rank test. **(C, D)** Box-and-whisker plots showing NCL mRNA expression levels in patient samples stratified by clinical characteristics. (C) NCL expression by high-risk status (no *vs* yes) in a 498-patient NB cohort. (D) NCL expression by recurrence or progression status (no *vs* yes) in an 88-patient NB cohort; samples with unknown or unavailable status were excluded. *p* values were determined using one-way ANOVA. Exact *p* values are reported unless *p* < 0.001; values below this threshold are shown as “*p* < 0.001”. **(E)** Forest plots showing hazard ratios (log_2_ scale) of NCL expression in relation to patient outcomes in the TARGET-NBL cohort. Top: high-risk and/or progressive or relapsed disease. Bottom: high-risk relapsed tumors with undifferentiated or poorly differentiated histology. Increasing hazard ratios across biologically aggressive subsets indicate progressive enrichment of NCL-associated risk. Hazard ratios and 95% confidence intervals are indicated, with associated *p* values. Exact *p* values are reported unless *p* < 0.001; values below this threshold are shown as “*p* < 0.001”. **(F)** Schematic summary of the *in vivo* experimental design. IMR-32 NCL^high^/CD34^+^ NB cells were inoculated subcutaneously into NOD/SCID mice. Fourteen days after tumor cell implantation, mice were randomized into vehicle control (Ctrl) or salinomycin (Sal; 5 mg/kg) treatment groups, and dosing was performed intraperitoneally once every three days for 4 weeks. **(G)** Tumor growth curves of IMR-32 xenografts treated with vehicle control (Ctrl) or salinomycin (Sal). Tumor volume was measured twice a week. Data are shown as mean ± standard error of the mean (*n* = 6 per group); statistical significance was determined by two-way ANOVA followed by Bonferroni-corrected post hoc testing. Compared with vehicle control (Ctrl): ∗∗*p* < 0.01. **(H)** Body weight curves during treatment demonstrated no significant difference between groups (*n* = 6 per group), indicating the tolerability of salinomycin at the administered dose. **(I)** Representative hematoxylin–eosin staining of xenograft tumors from control and salinomycin-treated groups at 20× and 100× magnification, with scale bars indicated. **(J)** Representative immunohistochemistry staining for NCL, nestin, c-Kit, and CD34 in control and salinomycin-treated tumors at 20× and 100× magnification. Rabbit IgG negative controls are shown in Figure S2. Scale bars: 200 μm (20× ) and 50 μm (100× ). **(K)** Quantification of immunohistochemistry staining in control versus salinomycin-treated xenografts. Percent positive staining area per field was quantified for NCL, nestin, and c-Kit, while CD34^+^ cells were quantified by the number of cells per field using ImageJ. For each indicated group, 20 fields of view were quantified across tumor sections from 6 mice. Each data point represents one quantified field of view, with the median and 25th/75th percentiles indicated. Statistical comparisons were performed using the unpaired Wilcoxon test. Exact *p* values are reported unless *p* < 0.001; values below this threshold are shown as “*p* < 0.001”. Statistical significance annotations indicate the actual significance levels versus the control group: ∗∗*p* < 0.01 and ∗∗∗∗*p* < 0.0001.

In our earlier work on CSC-targeting strategies in NB, we investigated salinomycin, a compound originally identified as a potent anti-CSC agent in breast cancer (∼100-fold more effective than paclitaxel). Mechanistic studies in NB identified NCL as a functional cellular binding target of salinomycin, which suppresses CSC marker expression by disrupting NCL occupancy at promoters of CSC-associated marker genes, including CD34.^4^ These findings established a direct mechanistic link between NCL and pharmacologic CSC targeting in NB. However, the *in vivo* relevance of NCL in NB remains undefined, representing a critical gap addressed in the present study.

To further evaluate whether pharmacologic targeting of NCL by salinomycin exerts anti-tumor effects *in vivo*, we conducted xenograft experiments using IMR-32 cells, which express CSC markers such as CD34, c-Kit, and nestin, and display high NCL expression. IMR-32 cells were implanted subcutaneously into 6-week-old NOD/SCID mice, followed by randomization into two treatment groups at two weeks post-inoculation. Salinomycin (5 mg/kg) was administered intraperitoneally once every three days for four weeks (Fig. 1F). Compared with controls, salinomycin significantly suppressed tumor growth (Fig. 1G) without detectable effects on body weight or overt clinical signs of toxicity (Fig. 1H). Histologic evaluation revealed high mitotic activity in control tumors and marked morphologic alterations in treated tumors, including increased necrosis, apoptosis, and differentiation (Fig. 1I). NCL immunostaining in control tumors was heterogeneous across cells, with prominent nuclear, cytoplasmic, and membrane staining, whereas salinomycin treatment reduced this heterogeneity and shifted NCL localization predominantly to the nucleus (Fig. 1J). Furthermore, salinomycin-treated tumors showed markedly reduced expression of nestin and c-Kit, together with fewer CD34^+^ tumor cells (Fig. 1J and K). These findings provide *in vivo* phenotypic evidence that pharmacologic targeting of NCL-driven cancer stemness suppresses NB tumor growth.

This study combines multiple patient cohorts and preclinical xenograft models to highlight the clinical significance of NCL in aggressive high-risk NB. Using a reduced-intensity salinomycin regimen, we demonstrate anti-tumor activity with suppression of CSC-associated features *in vivo*, supporting pharmacologic disruption of the NCL axis. Further clinical translation of NCL-targeting strategies will require validation in patient-derived and low-NCL models to strengthen generalizability and causal inference, as well as broader evaluation across NB-relevant molecular markers and functional pathways, together with the development of rationa l combination approaches designed to address tumor heterogeneity, disease progression, and relapse.

## CRediT authorship contribution statement

**Dan Qi:** Writing – review & editing, Writing – original draft, Visualization, Methodology, Investigation, Funding acquisition, Formal analysis, Data curation, Conceptualization. **Ronghu Ke:** Writing – review & editing, Writing – original draft, Methodology, Investigation, Formal analysis, Data curation. **Euni Wu:** Software. **Yizhong Pan:** Writing – review & editing. **S. Stephen Yi:** Writing – review & editing. **Jason H. Huang:** Writing – review & editing, Resources, Funding acquisition. **Erxi Wu:** Writing – review & editing, Supervision, Resources, Project administration, Methodology, Investigation, Funding acquisition, Conceptualization.

## Ethics declaration

All animal studies were conducted at a certified contract research organization (CRO) facility with Office of Laboratory Animal Welfare (OLAW) assurance.

## Data availability

Data are available from the corresponding author upon reasonable request.

## Funding

This work was supported by the 10.13039/100014109Baylor Scott & White Health Foundation (USA), the Corbett Estate Fund for Cancer Research (USA) (No. 62285-531021-41800, 62285-531021-61800 to Erxi Wu), the 10.13039/100000054National Cancer Institute (USA) (No. R21CA267885 to Erxi Wu), and, in part, by the Cancer Prevention and Research Institute of Texas, USA (No. RP240537 to Erxi Wu).

## Conflict of interests

Erxi Wu is a member of *Genes & Diseases* Editorial Board. He was excluded from all editorial decision-making related to the acceptance of this article for publication. Other authors declare no competing interests.
