# Supplementary material for: Targeting nucleolin-driven cancer stemness suppresses high-risk neuroblastoma in vivo
Source: Genes Dis. 2026 Jun 6;14(1):102278. doi: 10.1016/j.gendis.2026.102278 (PMC13553506; doi:10.1016/j.gendis.2026.102278)
Supplement: Supplementary file 1 [file mmc1.docx]

**Supplemental Materials**

**Materials and Methods**

**External Datasets**

We queried the R2: Genomics Analysis and Visualization Platform (https://r2.amc.nl) for publicly available neuroblastoma (NB) patient datasets. The TARGET-NBL cohort^1^ was used for survival, expression, and Cox regression analyses. In addition, we analyzed additional datasets, including a 498-patient NB cohort^2^ (Gene Expression Omnibus (GEO) accession number GSE62564) and an 88-patient NB cohort^3^ (GEO accession number: GSE16476) to further validate associations between NCL expression and NB clinical tumor stages and to perform Cox regression analyses.

Patients were stratified into high- and low-expression groups based on the median NCL expression level within each dataset. Overall survival (OS) was analyzed using the Kaplan–Meier method. Statistical significance between survival curves was assessed using the log-rank (Mantel–Cox) test. Hazard ratios (HRs) and 95% confidence intervals were calculated using Cox proportional hazards models, and *p* values were assessed by the Wald test. Multivariate Cox regression analyses were performed as indicated to evaluate the independent prognostic value of NCL relative to MYCN, ALK, and TERT expression. Correlation analyses between NCL and selected genes were performed using Pearson correlation coefficients (r), and group comparisons were evaluated using analysis of variance (ANOVA) where appropriate.

**Cell Culture**

IMR-32 NB cells were maintained *in vitro* with Minimal Essential Medium (MEM) supplemented with 0.01 mM Non-Essential Amino Acids (NEAA) and 10% fetal bovine serum (FBS) at 37 ºC in an atmosphere of 5% CO_2_ in air. The cells in exponential growth phase were harvested and quantified using a cell counter before tumor inoculation.

**Animal Study**

All procedures were reviewed and approved by the Institutional Animal Care and Use Committee (IACUC) of Crown Bioscience, Inc. prior to study initiation and were performed in accordance with Association for Assessment and Accreditation of Laboratory Animal Care (AAALAC) guidelines. Female NOD/SCID mice were housed in a specific pathogen–free facility with controlled temperature and humidity, a 12-hour light/dark cycle, and ad libitum access to autoclaved chow and water.

For tumor establishment, mice were inoculated subcutaneously in the right flank with 1 × 10^7^ IMR-32 cells in 0.2 mL phosphate-buffered saline (PBS) mixed 1:1 with Matrigel. Tumor growth was monitored twice weekly until 14 days after inoculation, when the mean volume reached ~194 mm^3^ and animals were randomized (day 0) into two treatment groups. Animals in the control group received the vehicle solution alone (10% dimethyl sulfoxide, 40% polyethylene glycol-300, 5% Tween-80, and 45% normal saline) administered intraperitoneally once every three days. Salinomycin^4^ was formulated in the same vehicle solution and administered intraperitoneally at 5 mg/kg^5^ on the same dosing schedule; control mice received vehicle only. Mice were examined daily for morbidity and signs of distress, including reduced activity, grooming abnormalities, hunched posture, dehydration, or labored breathing; body weight was recorded daily. Tumor dimensions were measured twice weekly with digital calipers and volume was calculated as V = (L × W^2^)/2. Humane endpoints included the presence of any tumor axis (length or width) greater than 2 cm, ulceration, > 20% body weight loss, or severe clinical deterioration, at which point mice were humanely euthanized under deep anesthesia. At study termination, tumors were excised, measured, weighed, and processed for histology and immunohistochemistry.

**Hematoxylin and Eosin (H&E) Staining**

Paraffin-embedded tumor tissues were sectioned at 5 µm, mounted on charged slides, deparaffinized in xylene, and rehydrated through graded ethanol to distilled water. H&E staining was performed using the H&E Stain Kit (Abcam, ab245880) according to the manufacturer’s instructions with minor modifications. Sections were stained with Mayer’s hematoxylin (Lillie’s modification) for 1 min, rinsed in running tap water, and blued in bluing reagent for 10–15 s, followed by washing in distilled water. Sections were counterstained with modified alcoholic eosin Y for 1 min, dehydrated through three changes of absolute ethanol, cleared in xylene, and mounted with a permanent mounting medium. Slides were examined and imaged using an Olympus Microscope BX51 System.

**Immunohistochemistry (IHC) Staining and Quantification**

For IHC, antigen retrieval was performed in citrate buffer (10 mM, pH 6.0) by boiling in a microwave oven, followed by incubation at 95 °C for 10 min and cooling to room temperature. Endogenous peroxidase activity was quenched with 3% H₂O₂ for 10 min, and nonspecific binding was blocked with 5% BSA in PBS for 30 min. Sections were incubated overnight at 4 °C with primary antibodies diluted in 3–5% BSA/PBS: anti-NCL (Cell Signaling Technology, #14574; 1:200), anti-nestin (BioLegend, #841901; 1:50), anti–c-Kit (Abcam, ab32363; 1:50), and anti-CD34 (Abcam, ab81289; 1:50). After PBS washes, Boost IHC Detection Reagent, horseradish peroxidase (HRP) (Rabbit) (Cell Signaling Technology, #8114) was applied for 40 min, followed by PBS washes. Signal was developed using the SignalStain® DAB Substrate Kit (Cell Signaling Technology, #8059) according to the manufacturer’s protocol, and slides were rinsed, counterstained with hematoxylin, dehydrated, cleared, and coverslipped. Negative controls were performed using rabbit IgG isotype control (Santa Cruz Biotechnology, sc-3888) in place of the primary antibody. Images were captured via an Olympus Microscope BX51 System.

Quantitative analysis was performed in Fiji (ImageJ 2.16.0/1.54p, NIH) following color deconvolution of hematoxylin and 3,3′-diaminobenzidine (DAB) signals. For each mouse, 3–5 non-overlapping fields of view from images acquired at 20× magnification were selected from viable tumor areas, and similarly sized rectangular regions of interest were defined. For NCL, nestin, and c-Kit, a single global DAB threshold, established using representative control and salinomycin-treated sections, was applied to all images. Expression was quantified as the percentage of DAB-positive area in each analyzed field of view and used for comparison between groups. CD34^+^ CSCs were defined as tumor cells exhibiting distinct DAB staining with an identifiable nucleus and distinguishable from vascular endothelial cells based on morphology and localization. The number of CD34⁺ cells per field of view from images acquired at 20× magnification was counted and used for comparison between groups.

**Statistical Analysis and Plots**

Gene expression differences between groups were evaluated using one-way analysis of variance (ANOVA). Tumor volumes in control and treatment groups were analyzed using two-way ANOVA with Bonferroni correction for multiple time-point comparisons. Quantification of positive immunohistochemical staining was assessed using the unpaired Wilcoxon test. A p value < 0.05 (raw or adjusted, as indicated) was considered statistically significant. Exact *p* values are reported unless *p* < 0.001; values below this threshold are shown as “*p* < 0.001”. All analyses and plots were generated in R (version 4.4.3 or 4.5.2) using the ggplot2, ggpubr, and rstatix packages within RStudio (2026.01.0, Build 392).

**Supplemental Results**

**NCL captures a distinct prognostic signal beyond established molecular markers in neuroblastoma**

To evaluate the prognostic significance of NCL in NB, we analyzed multiple publicly available datasets with annotated clinical outcomes, including overall survival (OS). Multivariable Cox regression analysis was performed incorporating NCL, MYCN, ALK, and TERT expression, enabling assessment using readily accessible transcriptomic measures rather than genomic alterations. In the 498-patient cohort, elevated NCL expression was strongly associated with inferior survival (HR = 5.8, *p* < 0.001), and this effect remained robust after multivariable adjustment, indicating that its prognostic value is not explained by these established molecular markers (**Fig. S1B**).

Our prior work reported that NCL expression is modestly elevated in MYCN-amplified NB and is associated with adverse clinical features and poor patient outcomes.^6^ Consistent with this, analysis of the TARGET NB cohort demonstrated that, in high-risk, progressive, or relapsed disease subsets (n = 67), NCL expression was positively associated with MYCN amplification status (*p* < 0.001; **Fig. S1C**, **left panel**), whereas correlations with ALK and TERT were weak (r < 0.3, data not shown), indicating limited collinearity. In this subgroup, multivariable Cox regression demonstrated that elevated NCL expression remained independently associated with adverse outcome (HR = 1.8, *p* < 0.001; **Fig. S1C**, **right panel**), consistent with univariate analysis (**Fig. 1E upper panel**). ALK also contributed to risk (HR = 1.8, *p* < 0.001), whereas TERT was not significantly associated with OS (**Fig. S1C**, **right panel**).

NCL expression was further enriched in biologically aggressive disease, including high-risk, progressive, or relapsed patients with undifferentiated or poorly differentiated tumors. In this subset (n = 53), NCL expression remained positively associated with MYCN amplification status (*p* < 0.001; **Fig. S1D, left panel**). Univariate Cox analysis demonstrated a strong association between elevated NCL expression and adverse outcome (HR = 3.1; *p* = 0.0018, **Fig. 1E lower panel**). After multivariable adjustment for ALK and TERT, the effect size of NCL was slightly attenuated but remained statistically significant (HR = 2.6, *p* < 0.001), ALK also contributed to risk in this subgroup (HR = 1.9, *p* < 0.001), whereas TERT again showed no significant association with OS (**Fig. S1D, right panel**).

An independent study using flow cytometry reported NCL positivity in approximately 50% of non–MYCN-amplified tumors and ~60% of MYCN-high tumors,^7^ supporting a modest association with MYCN while also indicating roles in both MYCN-dependent and MYCN-independent contexts.

Collectively, these findings demonstrate that NCL is a robust and independent prognostic biomarker in NB. The modest correlation with MYCN, together with persistent statistical significance after multivariable adjustment across cohorts and biologically defined subgroups, supports that NCL captures prognostic information that is partially shared with MYCN and largely independent of other established molecular markers, including ALK and TERT.

**Supplemental Figures**

**Figure S1. High NCL expression is associated with advanced tumor stage and poor overall survival in NB.**

**(A)** Association between NCL expression (log2 mean levels) and INSS stage in a 498-patient NB cohort. Bubble plot depicting NCL expression across International Neuroblastoma Staging System (INSS) stages; bubble size reflects the number of patients per stage, and color denotes relative NCL expression (scale at right). Differences among stages were evaluated by one-way ANOVA (*p* = 9.07 × 10^-10^).

**(B)** Multivariate Cox proportional hazards regression analysis in a 498-patient NB cohort evaluating the prognostic significance of NCL expression compared with expression levels of established NB risk markers, including MYCN, ALK, and TERT. Elevated NCL expression was strongly associated with poor survival (HR = 5.8, 95% CI 4.0–8.5, Wald *p* = 1.30 × 10^-19^), indicating a stronger association with survival than that of MYCN, ALK, or TERT expression.

**(C)** Correlation analysis in the TARGET NB dataset restricted to high-risk, progressive, or relapsed cases (upper panel of Figure 1E) demonstrates a positive association between NCL and MYCN amplification status (ANOVA *p* = 8.53 × 10^-8^), while correlations with ALK and TERT expression remain weak (r < 0.3, data not shown), indicating limited collinearity. Multivariate Cox proportional hazards regression analysis in the same cohort, incorporating NCL, ALK, and TERT expression, shows that elevated NCL expression remains independently associated with adverse outcomes (HR = 1.8, 95% CI 1.2–2.9, Wald *p* = 8.30 × 10^-3^), alongside contributions from ALK (HR = 1.8, 95% CI 1.4–2.4, Wald *p* = 1.40 × 10^-5^), whereas TERT is not significantly associated with survival in this subgroup. Exact *p* values are reported unless *p* < 0.001; values below this threshold are displayed as “*p* < 0.001”.

**(D)** Correlation analysis in the TARGET NB dataset focusing on high-risk, progressive, or relapsed tumors with undifferentiated or poorly differentiated (stem-like) features (lower panel of Figure 1E) similarly demonstrates a significant association between NCL and MYCN amplification status (ANOVA *p* = 3.42 × 10^-7^), with weak correlations observed between NCL and ALK or TERT expression (r < 0.3, data not shown). Multivariate Cox proportional hazards regression analysis in this aggressive subset further demonstrates that elevated NCL expression remains an independent predictor of poor survival (HR = 2.6, 95% CI 1.6–4.5, Wald *p* = 3.20 × 10^-4^), together with ALK (HR = 1.9, 95% CI 1.4–2.6, Wald *p* = 4.30 × 10^-5^), while TERT is not significantly associated with outcome in this subgroup. Exact *p* values are reported unless *p* < 0.001; values below this threshold are displayed as “*p* < 0.001”.

**Figure S2. Rabbit IgG negative control immunohistochemistry.**

Representative negative control staining of NB xenograft tumor sections incubated with normal rabbit IgG instead of primary antibody, shown at 20× (left) and 100× (right) magnification. No specific positive staining is observed, confirming the specificity of primary antibody staining in Figure 1. Scale bars: 200 μm (20×) and 50 μm (100×).

**References**

1.	Pugh, T.J. *et al.* The genetic landscape of high-risk neuroblastoma. *Nat Genet* **45**, 279–84 (2013).

2.	Su, Z. *et al.* An investigation of biomarkers derived from legacy microarray data for their utility in the RNA-seq era. *Genome Biol* **15**, 523 (2014).

3.	Molenaar, J.J. *et al.* Sequencing of neuroblastoma identifies chromothripsis and defects in neuritogenesis genes. *Nature* **483**, 589–93 (2012).

4.	Qi, D. *et al.* Salinomycin as a potent anticancer stem cell agent: State of the art and future directions. *Med Res Rev* **42**, 1037–1063 (2022).

5.	Yu, W.H. *et al.* Matrix Metalloprotease-7 Mediates Nucleolar Assembly and Intra-nucleolar Cleaving p53 in Gefitinib-Resistant Cancer Stem Cells. *iScience* **23**, 101600 (2020).

6.	Wang, F. *et al.* Nucleolin Is a Functional Binding Protein for Salinomycin in Neuroblastoma Stem Cells. *J Am Chem Soc* **141**, 3613–3622 (2019).

7.	Bensa, V. *et al.* Patient-derived neuroblastoma models are sensitive to nucleolin-recognizing liposomal drugs. *Biomed Pharmacother* **193**, 118817 (2025).
